# Recombinant human type-5 adenovirus (H101) combined with radiofrequency ablation versus traditional radiofrequency ablation for hepatocellular carcinoma ≤3 cm: a phase III randomized controlled trial

**DOI:** 10.1097/JS9.0000000000004438

**Published:** 2026-05-20

**Authors:** Hai-Su Dai, Zhi-Peng Liu, Hua-Qiang Bi, De-Hong Tan, Yu-Le Luo, Xian-Yu Yin, Yi Gong, Kuan-Sheng Ma, Zhi-Yu Chen, Kai Feng

**Affiliations:** Department of Hepatobiliary Surgery, Southwest Hospital, Army Medical University, Chongqing, China

**Keywords:** disease-free survival, hepatocellular carcinoma, randomized controlled trial, recombinant human type-5 adenovirus, RF ablation

## Abstract

**Background:**

Radiofrequency ablation (RFA) is standard for small hepatocellular carcinoma (HCC), yet recurrence remains common. This phase III trial compared RFA combined with recombinant human type-5 adenovirus (H101) versus traditional RFA in HCC ≤3 cm.

**Methods:**

This study prospectively collected data from patients between 2018 and 2021. This study included patients with a clinically or pathologically confirmed diagnosis of HCC characterized by a solitary tumor with a maximum diameter of ≤3 cm and excluded patients with distant metastasis. All patients were randomly allocated at a 1:1 ratio to the experimental group or the control group. The experimental group received RFA combined with H101, whereas the control group received traditional RFA. The primary endpoint was disease-free survival (DFS). The secondary endpoints included overall survival (OS) and safety. DFS and OS were evaluated in the intention-to-treat (ITT) population.

**Results:**

A total of 162 patients with small HCC were enrolled, with 81 patients randomized to each group, and ITT analysis was performed. The median follow-up durations for the RFA + H101 group and the RFA group were 36.47 and 37.57 months, respectively. The 3-year OS rates for the RFA + H101 group and the RFA group were 93.00 and 92.90%, respectively, and the 3-year DFS rates were 54.00 and 41.20%, respectively. Comparisons of OS rates and RFS rates between the two groups revealed no significant differences (OS: *P* = 0.688; DFS: *P* = 0.090). The safety profiles of the two groups were both high, with no statistically significant differences observed.

**Conclusion:**

Compared with traditional RFA, the combination of RFA with H101 did not result in a significant improvement in DFS rates among patients with small HCC. However, combination therapy has potential therapeutic advantages.

## Introduction

Hepatocellular carcinoma (HCC) ranks sixth in global cancer incidence and is the third leading cause of cancer-related mortality^[^1-3^]^. Advances in diagnostic techniques have led to an increased detection rate of small HCC (single lesion diameter ≤ 3 cm), encompassing both original and recurrent cases^[^4-7^]^. Given the limited availability of donor livers, the extensive use of liver transplantation for the treatment of small HCC is not realistic^[^8-11^]^. Surgical resection and radiofrequency ablation (RFA) are more frequently employed as treatment options for small HCC^[^12-15^]^. Our previous study demonstrated that long-term survival rates following surgery and RFA for small HCC patients are comparable; however, tumor residue after RFA appears to be more prevalent^[^16^]^. Therefore, reducing tumor residue post-RFA and enhancing the oncological benefits of RFA are critical for improving outcomes in patients with small HCC.

Recombinant human type-5 adenovirus (H101) is a genetically engineered adenovirus lacking the E1B 55 kDa protein that normally binds to p53 within cells to inhibit its function^[^17^]^. Owing to this deletion mutation, H101 cannot suppress p53 activity. Upon infection of tumor cells with mutant p53, H101 can replicate within these cells and exert oncolytic effects. Following cell death, the released virus infects adjacent tumor cells, further promoting tumor destruction^[^18,19^]^. Since the early 21st century, adenoviruses have been utilized in the treatment of HCC, including in combination with transarterial chemoembolization (TACE), for treating HCC patients with portal vein tumor thrombus^[^20,21^]^. However, as of the registration date of this study, no reports exist on the local treatment of HCC via RFA combined with H101.

In response to the increasing incidence of small HCC and the low disease-free survival (DFS) rate following RFA, we designed this prospective randomized controlled trial to compare the efficacy and safety of RFA combined with H101 versus traditional RFA in treating small HCC. This study aimed to explore strategies to reduce the risk of disease progression or recurrence after RFA for small HCC. Additionally, considering the significant differences in tumor biological characteristics and the immune microenvironment between original and recurrent HCC, we conducted subgroup analyses for original and recurrent HCC. This work has been reported in line with the TITAN criteria^[^22^]^.

## Methods

### Patient selection

Patients who were diagnosed with HCC from December 2018 to October 2021 were included in this study. The inclusion criteria were as follows: (1) clinically or pathologically confirmed diagnosis of HCC; (2) a single tumor with a diameter ≤3 cm; (3) there was no evidence of portal vein tumor thrombus or hepatic vein tumor thrombus at any level; (4) liver function classified as Child‒Pugh A or B; (5) indocyanine green retention at 15 min ≤30% and vital organ functions adequate to tolerate RFA or partial hepatectomy; (6) no obvious bleeding tendency: platelet count > 50 × 10^9^/l and prolonged prothrombin time < 5 s; (7) age between 18 and 70 years; and (8) no prior antitumor treatments before surgery. The exclusion criteria were as follows: (1) severe portal hypertension, history of upper gastrointestinal bleeding, or severe hypersplenism; (2) extrahepatic metastasis or lymph node metastasis; (3) multiple liver tumors detected by imaging or intraoperatively; (4) postprocedure pathological examination indicating non-HCC; and (5) patients who desired liver transplantation. The quality of this study was assessed using the Consolidated Standards Of Reporting Trials 2025 checklist^[^23^]^.

**Figure 1.**
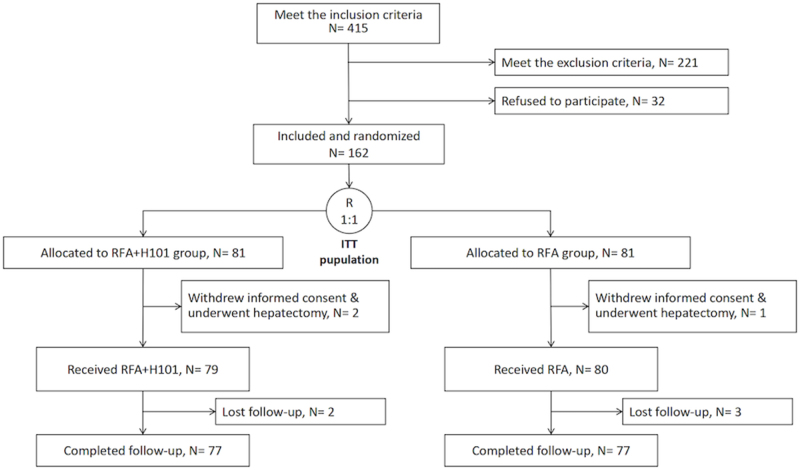
CONSORT flow diagram. RFA, radiofrequency ablation.

The participant flow diagram, illustrating the complete patient enrollment and exclusion process, is presented in accordance with the CONSORT guidelines (Fig. 1). This study has been registered at ClinicalTrials.gov (NCT03790059).

### Study design and sample size

This is a prospective randomized controlled trial, and the objective of this trial was to evaluate the short-term and long-term outcomes of RFA combined with H101 versus traditional RFA for treating HCC lesions ≤3 cm in diameter. This study adhered to the Declaration of Helsinki and received ethical approval. All patients provided informed consent. The protocol for this study is provided in the supplementary materials.

**HIGHLIGHTS**

- Compared to conventional RFA, RFA combined with H101 did not demonstrate significant superiority in treating single HCC lesions with a tumor diameter ≤3 cm, though a statistically marginal trend was observed (*P* = 0.090), providing preliminary data for future investigations.
- Both RFA combined with H101 and conventional RFA exhibited favorable safety profiles.

Patients were randomly assigned to the RFA + H101 group or the RFA group at a 1:1 ratio. On the basis of our previous reports, the 3-year recurrence rate for patients with small HCC after RFA treatment is approximately 30%^[^16^]^. Assuming a target 3-year recurrence rate of ≤12% for HCCs ≤3 cm after RFA + H101, with a significance level of *α* = 0.05 and power of 1 − *β* = 0.8, the required sample size was calculated via PASS 2008 software, resulting in 77 cases per group. Considering a potential 5% loss to follow-up, the adjusted sample size was 81 patients per group.

### Randomization

This study did not implement blinding. When a patient diagnosed with HCC at this center met the inclusion criteria, the multidisciplinary tumor team reviewed the patient’s data and determined eligibility. Upon confirmation of inclusion, randomization was initiated, and informed consent was obtained. The randomization sequence was computer-generated and placed into opaque, securely sealed envelopes by a designated research assistant. After patients signed the informed consent form, under the joint supervision of the trial inspector, research assistant, and surgeon, the envelopes were opened according to the patient sequence number to determine the surgical method. The surgeon then informed the patient about the trial details and treatment plan, initiating the treatment procedure.

### Radiofrequency ablation

In the RFA group, procedures under monitored anesthesia were performed via the LDRF-120S RFA system (Mianyang Leader Electronic Technology Co., Ltd, China) with LDDJS3-0200300A electrodes (Mianyang Leader Electronic Co., Ltd, China). The protocol included: (1) Routine disinfection; (2) Preoperative computed tomography (CT)/MRI imaging to optimize puncture paths, avoiding critical structures; (3) Intraoperative ultrasound-guided tumor assessment to determine electrode numbers. Ablation progressed sequentially from deep to superficial tumor regions; (4) Electrode insertion under real-time ultrasound guidance with continuous monitoring of ablation cycles and range adjustment based on tumor size; (5) Post-ablation needle tract coagulation followed by immediate CT confirmation of complete ablation and absence of bleeding/pneumothorax.

For the RFA + H101 group, after standard RFA completion and tumor cooling to <42°C, H101 (5.0 × 10^11^ vp/0.5 ml; two vials) was injected through the original puncture tract using a coaxial technique to avoid repeat puncture. An 18 G × 200 mm HAKKO SONOGUIDE PTC Type B needle was advanced coaxially along the radiofrequency electrode path for precise intratumoral and peritumoral injection.

### Endpoints and follow-up

The primary endpoint was DFS. The secondary endpoints included overall survival (OS), overall complications, major complications, abdominal infections, pulmonary infections, bile leakage, abdominal hemorrhage, and postoperative liver function. Data were collected by a clinical research assistant designated by the ethics committee and stored in a dedicated database management system. DFS was defined as the time from randomization to tumor-related disease progression, death, or loss to follow-up. The assessment of tumor-related disease progression was conducted according to the Response Evaluation Criteria in Solid Tumors version 1.1 (RECIST 1.1) criteria, with radiologists blinded to patient group assignments. OS was defined as the time from randomization to death or loss to follow-up.

According to the trial design, the duration from patient enrollment to trial completion is 3 years. The final statistical analysis of the entire trial was conducted after at least 12 months of follow-up. During the follow-up period, disease progression was the primary endpoint, and follow-up was continued until death was the secondary endpoint. Patients who died and patients with a follow-up period of 3 years were considered to have completed the trial. The follow-up requirements include tumor biomarker and liver function tests, along with enhanced CT scans every 2 months during the early postoperative period (within the first 6 months), followed by these examinations at 3-month intervals thereafter. All HCC patients underwent further therapeutic interventions following tumor recurrence or progression. The treatment modalities included but were not limited to repeat RFA, TACE, or systemic therapy. Comprehensive documentation of all administered treatment regimens was meticulously recorded (Supplemental Digital Content Table S1, available at: http://links.lww.com/JS9/G366).

### Statistical analysis

Statistical methods were selected on the basis of the nature of the trial data (measurement data, categorical data, ordinal data, and survival data). Categorical data were analyzed via the χ^2^ test or Fisher’s exact test. The measurement data were tested for normality and homogeneity of variance. Normally distributed variables are presented as the means ± standard deviations, whereas non-normally distributed variables are expressed as medians (ranges). To compare two-sample means, the *t*-test was used if assumptions were met; otherwise, the Wilcoxon rank sum test was applied. Survival curves were plotted via the Kaplan–Meier method, and differences between survival curves were examined via the log-rank test, with hazard ratios (HR) and 95% confidence intervals (CI) calculated. DFS and OS were analyzed in the intention-to-treat (ITT) population. Safety endpoints were evaluated in the safety set (SS) population. The ITT population was defined as all randomized individuals. The SS population was defined as all individuals who received at least one dose of the assigned treatment. A *P* value < 0.05 was considered statistically significant. Statistical analyses were conducted via SPSS 26.0 and R software (version 4.2.3; http://www.r-project.org/).

## Results

### Patient characteristics

A total of 415 HCC patients met the inclusion criteria, of whom 253 were excluded on the basis of the exclusion criteria. A total of 162 HCC patients were randomized between December 2018 and October 2021, with 81 patients allocated to each of the RFA + H101 groups and the RFA group. In the RFA + H101 group, two patients withdrew informed consent and underwent hepatectomy, while two were lost to follow-up. In the RFA group, one patient withdrew informed consent and underwent hepatectomy after randomization, whereas three patients were lost to follow-up. In the RFA + H101 group, 74 patients were male (91.4%), with an average age of 53.79 years, and 51 patients had recurrent HCC (63.0%). In the RFA group, 72 patients were male (88.9%), with an average age of 53.58 years, and 48 patients had recurrent HCC (59.3%). The baseline characteristics of the two groups were meticulously documented (Table 1). All patients underwent ultrasound contrast imaging immediately post-RFA, with no evidence of residual tumor activity observed in any case.

**Table 1:** Baseline characteristics of the two groups based on the intention-to-treat population.

Variables	RFA + H101 group (*N* = 81)	RFA group (*N* = 81)
Age, years^a^	53.8 ± 9.4	53.6 ± 9.4
Male	74 (91.4)	72 (88.9)
BMI, kg/m^2^^a^	23.8 ± 3.8	23.5 ± 3.1
Hepatitis B virus	81 (100)	80 (98.8)
ASA score		
Grade I	68 (84)	66 (81.5)
Grade II	12 (14.8)	14 (17.3)
Grade III	1 (1.2)	1 (1.2)
AFP, µg/l^a^	8.4 (3.1, 77)	7 (3.4, 52.9)
ALT, U/l^a^	25.2 (19, 34.1)	28.4 (19.8, 38.4)
ALB, g/l^a^	42 ± 4.7	41.2 ± 5.1
PLT, ×10^9^/l^a^	107 (71.5, 142.5)	98 (59.5, 149.5)
TBil, µmol/l^a^	16.3 (12.3, 22.2)	17.9 (12.1, 24.1)
Liver cirrhosis	81 (100)	78 (96.3)
Child‒Pugh grade		
Grade A	79 (97.5)	78 (96.3)
Grade B	2 (2.5)	3 (3.7)
ALBI grade		
Grade 1	57 (70.4)	54 (66.7)
Grade 2	22 (27.2)	25 (30.9)
Grade 3	2 (2.5)	2 (2.5)
Imaging tumor size, mm^a^	20 (16, 23)	18 (14, 21)
Type of HCC		
Naive HCC	30 (37)	33 (40.7)
Recurrent HCC	51 (63)	48 (59.3)

AFP, alpha fetoprotein; ALB, albumin; ALT, alanine aminotransferase; ASA, American Society of Anesthesiologists; BMI, body mass index; PLT, platelet; TBil, total bilirubin; RFA, radiofrequency ablation; HCC, hepatocellular carcinoma.

^a^ Continuous variables with normal distribution are presented as mean ± SD, and skewed variables as median (interquartile range, IQR).

### Long-term outcomes

**Figure 2.**
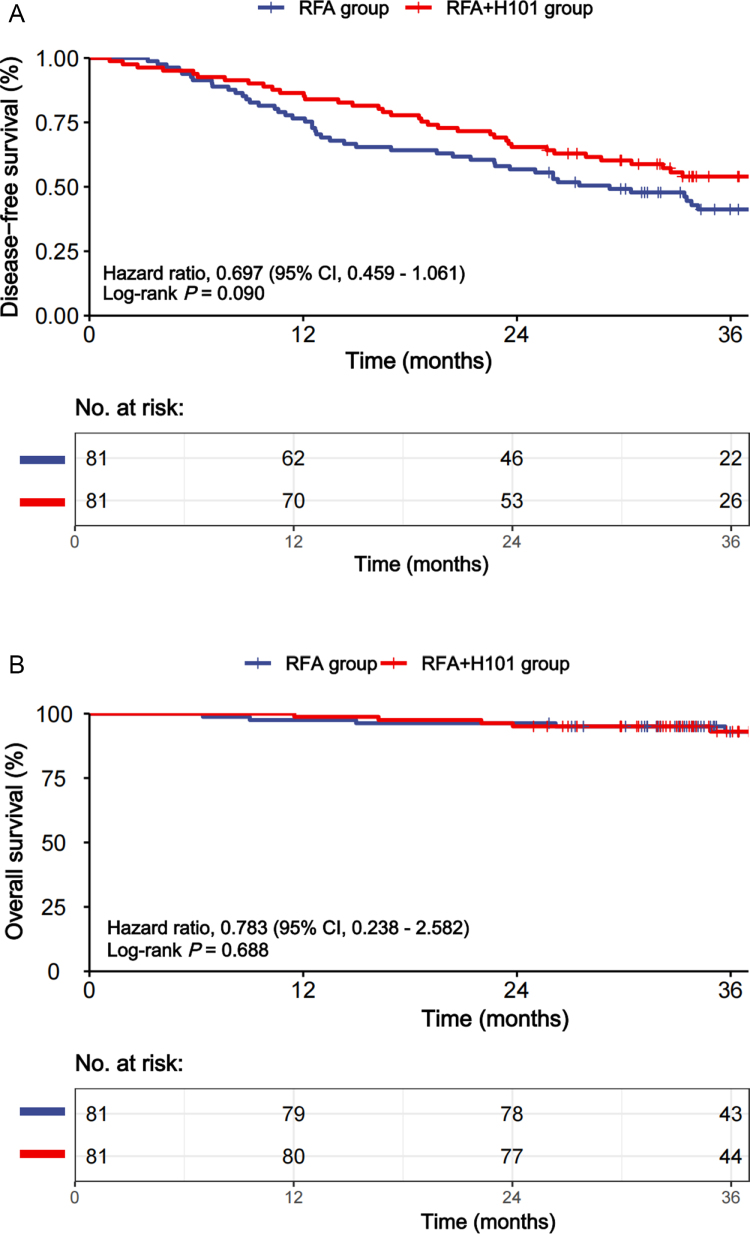
Disease-free survival (A) and overall survival (B) between the RFA + H101 group and the RFA group based on the intention-to-treat population. RFA, radiofrequency ablation.

Long-term outcomes were analyzed in the ITT population, with 81 patients in each group. The median follow-up durations for the RFA + H101 group and the RFA group were 36.47 (32.28, 49.03) months and 37.57 (32.97, 47.30) months, respectively. As of the final follow-up, 11 patients had died (all due to tumor progression). The 1-, 2-, and 3-year OS rates for the RFA + H101 group and the RFA group were 98.80, 95.10, and 93.00% and 97.50, 96.30, and 92.90%, respectively. Recurrence occurred in 89 patients, comprising 63 cases of early recurrence (<2 years) and 26 cases of late recurrence (≥2 years) (Supplemental Digital Content Table S2, available at: http://links.lww.com/JS9/G366). The 1-, 2-, and 3-year DFS rates were 86.40, 65.40, and 54.00% and 76.50, 56.80, and 41.20%, respectively. Comparisons of the DFS rates (Fig. 2A) and OS rates (Fig. 2B) between the RFA + H101 group and the RFA group revealed no significant differences (DFS: *P* = 0.090, HR: 0.697, 95% CI: 0.459–1.061; OS: *P* = 0.688, HR: 0.783, 95% CI: 0.238–2.582).

### Short-term outcomes

Short-term outcomes were analyzed in the SS population, with 79 patients in the RFA + H101 group and 80 patients in the RFA group. There were no treatment-related deaths in either group. The RFA group experienced complications, including abdominal infection (one patient), pulmonary infection (two patients), bile leakage (one patient), and abdominal hemorrhage (one patient). The RFA + H101 group experienced complications, including abdominal infection (one patient) and abdominal hemorrhage (one patient). Overall, complications occurred in 12 patients (15.2%) in the RFA + H101 group and 15 patients (18.8%) in the RFA group. Major complications occurred in four patients (5.1%) in the RFA + H101 group and three patients (3.8%) in the RFA group. No significant differences were observed in short-term outcomes between the two groups (Table 2).

**Table 2:** Short-term outcomes between the RFA + H101 group and RFA group on the basis of the safety set population.

Outcomes	RFA + H101 group (*N* = 79)	RFA group (*N* = 80)	*P* value
Overall complications (C–D grade I–IV)	12 (15.2)	15 (18.8)	0.550
Major complications (C–D grade III/IV)	4 (5.1)	3 (3.8)	0.986
Blood transfusion	0 (0.0)	0 (0.0)	1.000
Abdominal infection	1 (1.3)	1 (1.3)	0.482
Pulmonary infection	0 (0.0)	2 (2.5)	0.482
Biliary leakage	0 (0.0)	1 (1.3)	0.995
Abdominal hemorrhage	1 (1.3)	1 (1.3)	0.482
Unplanned readmission	0 (0.0)	0 (0.0)	1.000

RFA, radiofrequency ablation.

### Post hoc subgroup analyses

**Figure 3.**
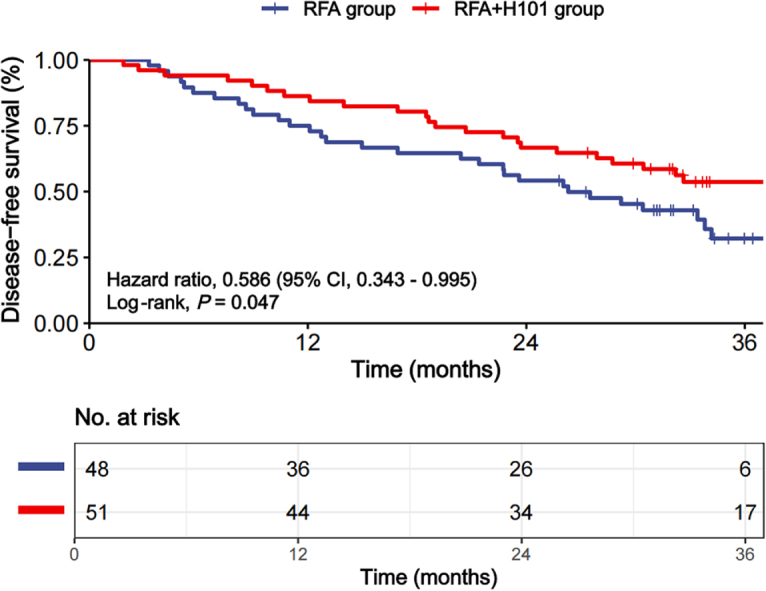
Disease-free survival between the RFA + H101 group and the RFA group in patients with recurrent HCC based on the intention-to-treat population. RFA, radiofrequency ablation; HCC, hepatocellular carcinoma.

This study conducted *post hoc* exploratory analyses comparing DFS rates between the RFA group and the RFA + H101 group in recurrent HCC patients, based on IIT subgroup classification. The analysis revealed that in patients with recurrent HCC, the RFA + H101 group had a significant improvement in DFS. Among the 99 patients with recurrent HCC, the median follow-up periods for the RFA + H101 group and the RFA group were 37.67 (32.97, 57.33) months and 34.17 (31.31, 37.63) months, respectively. The 1-, 2-, and 3-year DFS rates were 86.30, 66.70, and 53.70%, respectively, for the RFA + H101 group and 75.00, 54.20, and 32.20%, respectively, for the RFA group. In the recurrent HCC subgroup, the DFS rate of the RFA + H101 group was significantly greater than that of the RFA group (DFS: *P* = 0.047, HR: 0.586, 95% CI: 0.343–0.995) (Fig. 3).

## Discussion

This prospective randomized controlled trial evaluated the short-term and long-term outcomes of RFA combined with H101 compared with traditional RFA in treating HCC lesions ≤3 cm in diameter. The results demonstrated that both RFA combined with H101 and traditional RFA had low complication rates. Although RFA combined with H101 did not significantly improve the DFS or OS rates, there was a trend toward better DFS rates in the RFA + H101 group than in the traditional RFA group. To our knowledge, this is the first randomized controlled trial to investigate the long- and short-term effects of RFA combined with H101 for small HCC patients compared with traditional RFA.

The detection rate of small HCC (single lesion diameter ≤ 3 cm) is increasing. For patients with small HCC, liver transplantation offers the best long-term survival outcomes. However, owing to the scarcity of donors, the widespread application of liver transplantation for small HCC is not practical. Surgical resection can also provide long-term survival benefits, but even with the remarkable regenerative capacity of the liver, there are limitations to the extent to which liver resection can be safely performed^[^24,25^]^. Consequently, RFA has emerged as a first-line treatment for these patients when liver transplantation is not an option^[^26-30^]^. However, our previous studies have shown that while RFA and surgical resection yield similar survival rates for patients with small HCC, RFA is more likely to leave residual tumor tissue^[^16^]^. This may be attributed to several factors: (1) The ablation needle may not always cover the largest cross-sectional area of the tumor^[^31,32^]^. (2) If the tumor is near large blood vessels, the heat generated during ablation can be rapidly dissipated by blood flow^[^33,34^]^. (3) Although undetectable by imaging, microvascular invasion and microsatellite lesions around small HCCs may still exist^[^33,35-37^]^. These factors contribute to a greater risk of recurrence after RFA. To further improve the oncological outcomes of RFA, additional research is warranted.

Oncolytic viruses, as an emerging therapeutic strategy for cancer, have demonstrated significant potential in the treatment of HCC through their ability to specifically infect and lyse tumor cells^[^38^]^. Previous studies have shown that H101 combined with TACE can improve OS and progression-free survival in patients with unresectable HCC^[^21,39^]^. Additionally, research has confirmed the efficacy of H101 in treating HCC with portal vein tumor thrombus^[^40^]^. Furthermore, the combination of oncolytic viruses with chemotherapeutic agents such as cisplatin, gemcitabine, paclitaxel, or vincristine has exhibited a strong synergistic effect on non-small cell lung cancer cells^[^41^]^. A prospective phase II clinical trial demonstrated that H101 combined with conventional chemotherapy can improve the prognosis of refractory malignant tumors, with low toxicity and good tolerability^[^42^]^. Recently, a novel oncolytic virus, VG161, has been utilized for advanced HCC. VG161, an engineered oncolytic herpes virus expressing Interleukin-12, Interleukin-15, Interleukin-15 receptor alpha, and a PD-1/PD-L1-blocking fusion protein, was assessed in a multicenter phase 1 trial. The trial results indicated that VG161 is safe, with no dose-limiting toxicities observed, and showed promising efficacy. It can reshape the tumor immune microenvironment and re-sensitize tumors resistant to prior treatments^[^43^]^. However, to date, no clinical trials have explored the combination of oncolytic viruses with RFA for the treatment of HCC. To address this research gap, we designed this prospective randomized controlled trial to investigate the efficacy of RFA combined with H101 compared with traditional RFA in treating small HCC.

Although the results of this study did not confirm a significant benefit of H101 combined with RFA over traditional RFA in the treatment of small HCC, the findings suggest potential advantages. This potential may be attributed to several factors: (1) Mechanism of action of H101. H101 is a genetically engineered adenovirus lacking the E1B 55 kDa protein, which normally binds to p53 within cells to inhibit its function^[^21,39,44,45^]^. Owing to this deletion mutation, H101 cannot suppress p53 activity. Upon infection of tumor cells with mutant p53, H101 can replicate within these cells and exert oncolytic effects^[^46^]^. After cell death, the released virus infects adjacent tumor cells, further promoting tumor destruction^[^47^]^. (2) Intratumoral injection facilitates drug accumulation. During the treatment process, we chose intratumoral injection of H101. If intravenous administration was used, the normal liver’s uptake of the virus would significantly reduce the amount of virus reaching the tumor site. Additionally, the humoral immune response and the presence of neutralizing antibodies against adenovirus in the serum further restrict viral proliferation and spread. In contrast, intratumoral injection facilitates the accumulation of the virus at the tumor site, thereby enhancing its therapeutic effect^[^21,47^]^.

In this study, we performed *post hoc* exploratory analyses revealing that the combination of RFA and H101 in patients with recurrent HCC demonstrated notable oncological benefits. This may be attributed to the distinct biological behaviors between naïve and recurrent HCC, combined with H101’s mechanism of action. As an oncolytic agent, H101 selectively lyses tumor cells to exert antitumor effects. The characteristic high invasive and proliferative activity of recurrent HCC provides a favorable microenvironment for H101. However, these findings originated from *post hoc* exploratory analyses, necessitating cautious interpretation of RFA + H101 benefits in recurrent HCC populations. This study has the following limitations: (1) All the data were collected from a single center, which may limit the generalizability of the results to other centers. (2) The sample size was relatively small. When the sample size was set, there was a lack of previous data on the combination of RFA and H101, leading to an insufficiently large calculated sample size. On the basis of the results of this study, we obtained more precise data. Future studies should include multicenter, adequately sized randomized controlled trials to explore the short-term and long-term outcomes of the combination of RFA and H101.

## Conclusion

In conclusion, this study is the first randomized controlled trial to explore the combination of RFA and H101 versus traditional RFA for small HCC. We found that both treatment approaches ensured comparable perioperative safety in the management of small HCC. Although the long-term survival benefit of RFA combined with H101 is limited, it has the potential to control tumor recurrence.

## Data Availability

The data used in this study were collected from author’s institution. All data were anonymized to ensure the privacy and confidentiality of the participants. For access to the data, please contact the corresponding author.

## Research registration unique identifying number (UIN)

This study has been registered at ClinicalTrials.gov (NCT03790059).
